# ORi™: a new indicator of oxygenation

**DOI:** 10.1007/s00540-021-02938-4

**Published:** 2021-04-26

**Authors:** Yusuke Ishida, Toshio Okada, Takayuki Kobayashi, Hiroyuki Uchino

**Affiliations:** Department of Anesthesiology, Tokyo Medical University, 6-7-1 Nishishinjuku, Shinjuku-ku, Tokyo, 160-0023 Japan

**Keywords:** Oxygenation reserve, Hypoxemia, Hyperoxia, Oxygen reserve index (ORi)

## Abstract

In the perioperative period, hypoxemia and hyperoxia are crucial factors that require attention, because they greatly affect patient prognoses. The pulse oximeter has been the only noninvasive monitor that can be used as a reference of oxygenation in current anesthetic management; however, in recent years, a new monitoring method that uses the oxygen reserve index (ORi™) has been developed by Masimo Corp. ORi is an index that reflects the state of moderate hyperoxia (partial pressure of arterial oxygen [PaO_2_] between 100 and 200 mmHg) using a non-unit scale between 0.00 and 1.00. ORi monitoring performed together with percutaneous oxygen saturation (SpO_2_) measurements may become an important technique in the field of anesthetic management, for measuring oxygenation reserve capacity. By measuring ORi, it is possible to predict hypoxemia and to detect hyperoxia at an early stage. In this review, we summarize the method of ORi, cautions for its use, and suitable cases for its use. In the near future, the monitoring of oxygen concentrations using ORi may become increasingly common for the management of respiratory function before, after, and during surgery.

## Introduction

Over the years, intraoperative anesthetic monitoring techniques have been improved similarly to the improvement of surgical instruments. Blood pressure, heart rate, electrocardiography, and blood oxygen saturation level are mainly monitored during intraoperative anesthesia. In the present article, we review the oxygen reserve index (ORi™), which is a measurement that enables the assessment of oxygen reserve capacity, and discuss its various applications and future outlooks.

## What is ORi

During anesthetic management, predicting the partial pressure of arterial oxygen (PaO_2_) is difficult when percutaneous oxygen saturation (SpO_2_) is 100%. In usual cases, in which arterial lines of the patients are secured, arterial blood gas analysis for the appropriate management of PaO_2_ is possible. However, as this is an invasive method, it cannot be performed on all patients. In most patients undergoing general anesthesia, tracheal intubation and artificial ventilation are carried out to manage positive-pressure ventilation. However, during surgery, atelectasis and pulmonary injury may occur owing to multiple factors [1–3], which may cause sudden hypoxemia during or after surgery, making it difficult to manage the patient [1, 4, 5]. Therefore, PaO_2_ during surgery is a useful indicator for anesthesiologists, particularly in patients with low respiratory function (e.g., obstructive/restrictive ventilatory disorder) and low oxygen reserve (e.g., pregnant women and obese patients) [6, 7]. In addition, controlling oxygen concentrations during surgery in patients with diseases in which hyperoxia should be avoided, such as interstitial pneumonia and retinopathy of prematurity [8], is a difficult problem. Although hypoxia can lead to adverse effects, hyperoxia during surgery can also induce pulmonary injury and absorption atelectasis, resulting in postsurgical respiratory complications [1–3]. Furthermore, hyperoxia can also increase the risk of other adverse events [9] (Table 1). Unfavorable clinical outcomes owing to a high fraction of inspired oxygen (FiO_2_) have been recently reported in critically ill adults, including patients with chronic obstructive pulmonary disease, myocardial infarction, cardiac arrest, stroke, and traumatic brain injury [10–15].

**Table 1:** Adverse events caused by exposure to high oxygen concentrations

Absorption atelectasis
Vasoconstriction
Central nervous system toxicity
Alveolar edema
Pulmonary fibrosis
Hyperoxic acute lung injury
Increase in reactive oxygen species
Increased mortality after resuscitation
Higher incidence of reinfarction after acute myocardial infarction
Increased cancer recurrence
Increased risk of fire during certain procedures
(Cited from Anesth Analg. 2017;125:682–687 and modified)

**Fig. 1:**
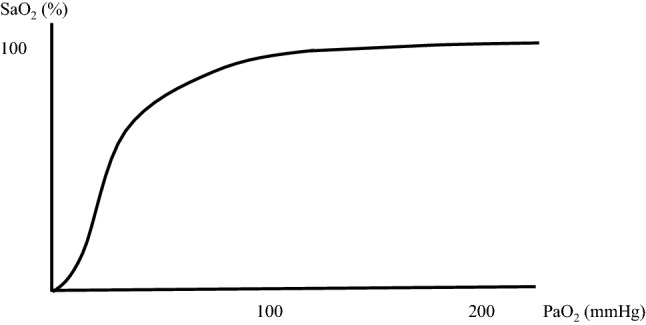
Correlation between PaO_2_ and SaO_2_. **a** The oxygen dissociation curve for hemoglobin is shown. A correlation is observed up until the PaO_2_ is 100 mmHg, but when SpO_2_ reaches 100%, it becomes impossible to estimate PaO_2_. *PaO*_*2*_ partial pressure of arterial oxygen, *SaO*_*2*_ arterial oxygen saturation

Until recently, the pulse oximeter was the only noninvasive instrument to measure arterial blood oxygen saturation (SaO_2_), which is an index of blood oxygenation. During the 1970s, the world’s first pulse oximeter that was able to measure SaO_2_ from the fingertip was commercialized. Since then, this device has been used in a wide range of situations for the noninvasive monitoring of blood oxygenation [16]. However, this device has its flaws. Although SpO_2_ and PaO_2_ are correlated at most points, when SpO_2_ reaches 100%, estimating PaO_2_ becomes difficult [17] (Fig. 1). Thus, other methods, such as arterial blood gas analysis, are often performed to measure PaO_2_ for the evaluation of oxygenation.

**Fig. 2:**
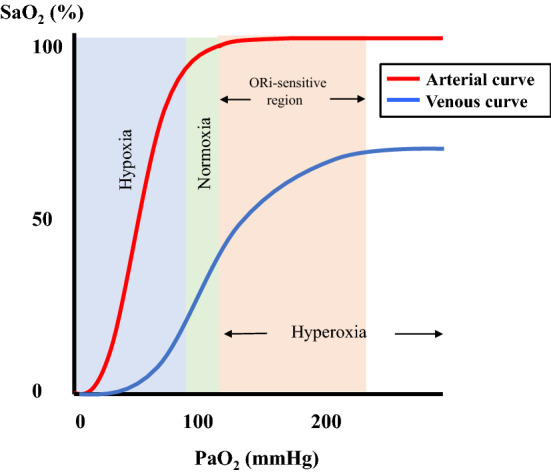
Mechanism of ORi.** a** There is a correlation between SpO_2_ and PaO_2_ up to a PaO_2_ of 100 mmHg, and by measuring the SpO_2_, it is possible to estimate PaO_2_. For PaO_2_ values of more than 100 mmHg, SpO_2_ will have reached a maximum and will not change any further; however, SvO_2_ will continue to increase. A change in SvO_2_ causes a change in the absorption of the incident light in parallel with changes in PaO_2_, resulting in changes in the measurement signal. Using the Rainbow SET technology established by Masimo Japan, it is possible to extract these measurement signals, enabling the detection of PaO_2_ changes through changes in SvO_2_. The SvO_2_ value reaches a plateau when PaO_2_ exceeds more than 200 mmHg. Thus, ORi reflects changes in PaO_2_ in the range of 100 to 200 mmHg (orange area). (Modified and reproduced with permission) (Springer Nature; J Clin Monit Comput) [19] http://creativecommons.org/licenses/by/4.0/. **b** Schematic representation of arterial (red line) and venous (blue line) oxyhemoglobin dissociation curves. *PaO*_*2*_ partial pressure of arterial oxygen, *SaO*_*2*_ arterial oxygen saturation, *SvO*_*2*_ venous oxygen saturation, *ORi* oxygen reserve index

**Fig. 3:**
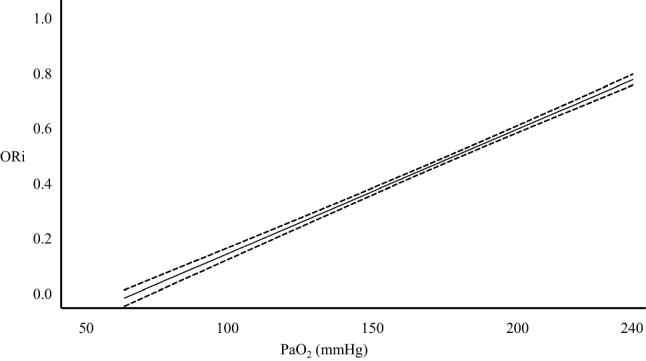
Correlation between PaO_2_ and ORi.** a** A positive correlation is observed between ORi and PaO_2_ below 240 mmHg (*r*^2^ = 0.536). The dotted lines indicate the 95% confidence interval of the regression line. (Modified and reproduced with permission) (Wolters Kluwer Health; Anesth Analg) [23]. *PaO*_*2*_ partial pressure of arterial oxygen, *ORi* oxygen reserve index

The oxygen reserve index (ORi) is a noninvasive and continuous parameter that can be measured by connecting a multi-wavelength sensor to Root^®^ with Radical-7^®^, Pulse CO-Oximeter^®^ (Masimo Corp., Irvine, CA, USA). ORi is an index measured using a non-unit scale between 0.00 and 1.00, which reflects the range of moderate hyperoxia (PaO_2_ between 100 and 200 mmHg), and is defined as oxygen reserve reflecting the level of venous blood oxygen saturation (SvO_2_). ORi remains at 0.00 in a room air environment. With supplemental oxygen, after SpO_2_ reaches 100%, SvO_2_ also increases as PaO_2_ does, and usually reaches a plateau at about a PaO_2_ of 200 mmHg [18, 19] (Fig. 2). In general, a pulse oximeter displays the SpO_2_ value measured from light absorption of the pulsatile blood at the site where the sensor is placed. Pulsatile changes are observed in the arteries, capillaries, and venules. Masimo developed rainbow SET^®^ which can analyze changes in the absorption of incident light of both arterial and venous blood using a multi-wavelength sensor, and display them as ORi, which is a unitless scale between 0.00 and 1.00. Thus, ORi begins to increase from 0.00 at about a PaO_2_ of 100 mmHg, and reaches a plateau of 1.00 at approximately 200 mmHg [18–21]. However, there are reports stating that in cases where FiO_2_ is 1.0, even when oxygenation is complete, the peak value is different for each individual and reaches a plateau at about 0.60 [22]. Applegate et al. analyzed the association between ORi and PaO_2_ during surgery, and found that if PaO_2_ is below 240 mmHg, ORi and PaO_2_ show a positive correlation (*r*^2^ = 0.536) [23] (Fig. 3). However, if PaO_2_ is higher than 240 mmHg, there was no correlation (*r*^2^ = 0.0016). Furthermore, when ORi was more than 0.24, PaO_2_ was more than 100 mmHg. As the report showed that 96.6% of the samples had a PaO_2_ of ≥ 150 mmHg when ORi was above 0.55, the authors concluded that ORi can be used as an indicator of PaO_2_. Vos et al. investigated the correlation between PaO_2_ and ORi during the administration of oxygen in 20 healthy volunteers, and they also reported that the two values have a strong positive correlation [24]. In addition, regarding the correlation between PaO_2_ and ORi during anesthesia under oxygenation, when PaO_2_ was less than 240 mmHg (*n* = 69), linear regression analysis showed a relatively strong positive correlation (*r*^2^ = 0.706) [25].

## Suitable cases for the use of ORi with regard to anesthetic management

The following is a list of cases for which the use of ORi is suitable.

- Rapid sequence induction

Normally in a room air environment, ORi is 0.00. After the administration of oxygen, even after SpO_2_ reaches 100%, ORi will continue to increase. Rapid sequence induction (RSI) is appropriate for patients in whom the aspiration of gastric contents may occur, including those with a full stomach, ileus, and expectant mothers [26–28]. In RSI, tracheal intubation is performed without ventilation after the occurrence of apnea. In such cases, ORi may be an indicator of the time it will take for SpO_2_ to decrease during tracheal intubation. Yoshida et al. investigated the usefulness of ORi during RSI [29]. They reported that in 10 out of 17 patients (77%) in whom preoxygenation was performed, ORi started to decrease 32.5 s (IQR: 18.1–51.3) before a decrease in SpO_2_ post-apnea. By monitoring the ORi, it is possible to predict a decrease in oxygenation about 30 s before SpO_2_ starts to decrease. From this finding, Yoshida concluded that it may be possible to lower the incidence rate of hypoxemia during RSI. Thus, ORi is expected to be useful for RSI.

In addition, owing to the recent outbreak of coronavirus disease 2019 (COVID-19), RSI has been recommended to prevent the infection of anesthesiologists during intubation [30, 31]. Furthermore, the use of aerosol boxes for the prevention of COVID-19 infection makes intubation difficult [32–34]. Thus, aerosol boxes may increase intubation times and therefore increase the risk of hypoxia in patients [32]. Using ORi may hence reduce the risk of hypoxemia and enable safe tracheal intubation.

- Pediatric applications

In pediatric patients, oxygen consumption is higher, and the closing volume is also relatively larger than the functional residual capacity compared with adult patients. Owing to this fact, the development of hypoxemia in pediatric patients is more rapid than in adult patients during apnea. Previous studies investigating ORi in pediatric patients during anesthetic induction showed that ORi monitoring enabled detection of a reduction in oxygenation approximately 30 s prior to a rapid change in SpO_2_ [21]. These studies suggest that prediction of the onset of a rapid decrease in oxygen saturation enables the prompt implementation of appropriate treatments, which is an advantage. In fact, the use of ORi for tracheoplasty in children was found to enable the earlier prediction of hypoxemia [35]. In addition, as the trachea is shorter in children than in adults, when performing tracheal intubation and head bending during surgery, there is a higher possibility of unilateral intubation in children [36, 37]. Therefore, in the former case, comparison of ORi before and after intubation may be useful, and in the latter case, a sudden drop in ORi during the operation may be a useful indicator of subsequent hypoxemia. Therefore, the use of ORi may enable the earlier implementation of appropriate treatments. All of the above demonstrate that ORi is a useful indicator in pediatric anesthesia during the perioperative period.

- Other suitable cases

ORi is also useful for patients in whom respiration management is required during one-lung ventilation (OLV). Hypoxemia may still occur in about 10% of such patients [38]. When switching from two-lung to OLV, the shunt fraction increases, oxygenation is impaired, and hypoxemia may occur [39]. Studies monitoring ORi during the anesthetic management of OLV showed that decreases in PaO_2_ could be detected much earlier than when only SpO_2_ was monitored. These studies suggest that ORi can reduce the risk of complications during OLV [40]. Another study reported that the monitoring of ORi during OLV could predict hypoxemia [41]. These findings may be helpful to adjust FiO_2_ individually in patients undergoing OLV, and to avoid unnecessarily high concentrations of oxygen.

In addition, the use of ORi is suitable for surgeries that have a high probability of leading to atelectasis, such as surgeries on obese patients [42, 43], surgeries in the Trendelenburg position, and laparoscopic surgeries [43–45]. Other studies also suggested that ORi is useful in situations in which awake intubation is performed [22]. ORi monitoring may be able to avoid hypoxemia in cases in which bronchial stenting using a rigid endoscope is performed while the patient continues to breathe spontaneously [46]. For surgeries in which patients’ respiratory statuses can vary, ORi monitoring can be an indicator to decide whether or not early intervention is required to avoid hypoxemia [47]. ORi monitoring during the induction of anesthesia can be an indicator of whether preoxygenation is being carried out appropriately.

In addition, hyperoxia can be detected by monitoring ORi in patients under general anesthesia, and thus the administration of unnecessarily high concentrations of oxygen can possibly be avoided. In fact, there are several reports demonstrating that the use of ORi in managing oxygen levels during and after surgery, and for patients in the intensive care unit (ICU) can prevent exposure to excess oxygen [25, 48, 49]. In general, high oxygen concentrations are more harmful to infants, children, and older patients than to adults [50]. Oxidative stress that occurs as a consequence of treatment with high oxygen concentrations during the perinatal period notably affects the lung and brain development of premature and newborn infants. For instance, bronchopulmonary dysplasia is caused by hyperoxic lung injury [51–53]. In older patients, the production and accumulation of reactive oxygen species (ROS) are largely increased, and antioxidative functions are decreased, resulting in various aging-associated diseases [54, 55]. Hyperoxia promotes ROS production, suggesting that perioperative hyperoxia might enhance the adverse effects of aging-associated diseases, particularly in lung and brain diseases. Therefore, adequate oxygenation using ORi may lead to a reduction in complications.

## Methods and cautions of the use of ORi

**Fig. 4:**
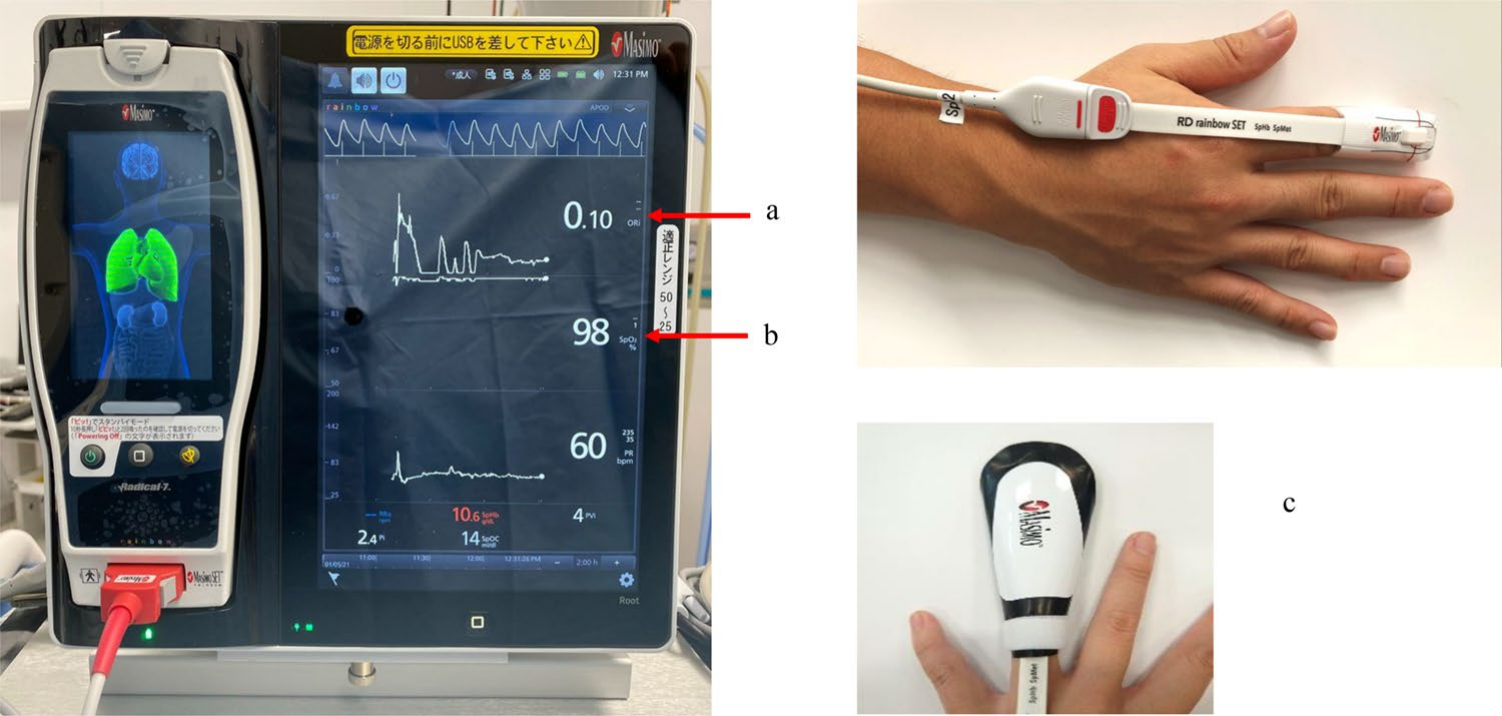
ORi function built into Root^®^ with Radical-7^®^. **a** ORi, **b** SpO_2_, **c** blockage of light is necessary to measure ORi using a SpHb sensor. *ORi* oxygen reserve index, *SpO*_*2*_ percutaneous oxygen saturation

ORi is measured by placing an RD rainbow Lite SET^®^ sensor, which is a multiwavelength sensor, on a patient’s fingertip and connecting it to Root^®^ with Radical-7^®^. In addition to ORi, this sensor can measure a patient’s SpO_2_, pulse rate (PR), Perfusion Index (Pi), and Pleth Variability Index (PVi^®^) (Fig. 4). In addition, ORi can be measured by a RD rainbow SET^®^-2 sensor, which can measure Noninvasive and Continuous Hemoglobin (SpHb^®^). However, it is necessary to block any outside light when using the SpHb sensor, as this sensor is easily affected by light owing to its high sensitivity to long wavelengths (Fig. 4). Furthermore, special attention is needed to make sure that the SpO_2_ is greater than 98% because if this criterion is not established, ORi does not show a positive number. One caution during monitoring is that measurement is difficult when the patient’s body is moving or when the blood perfusion rate is very low. Furthermore, ORi values may fluctuate if a pigmented drug is administered intravenously. There is a report showing that ORi rapidly decreased after indigo carmine was administered intravenously [56]. Another report showed that when indocyanine green (ICG) was administered to 10 patients, ORi increased in all of the patients [57]. The authors of this study suggested that it is hence important to take into account an increase in ORi when using ICG during surgery. ORi values can also fluctuate for other unknown reasons, and hence it is also important to make a comprehensive assessment of the patient’s condition and possible effects. In addition, the Radical-7 software version for the calculation of ORi and the RD rainbow Lite SET sensor were updated in January 2018, and although the peak is different for each individual, a plateau is reached at about 1.00 in most cases, and the artifact elimination function has been improved compared with the previous version. It is hence important to note that the obtained ORi values may differ depending on the version of the device used, so care must be taken when interpreting the results of previous studies.

## Outlooks of ORi

As mentioned above, in recent years, ORi has been used as an index of oxygenation during surgery. However, in the near future, ORi may also be used in situations in which changes in oxygenation occur frequently, such as in patients in the ICU and general wards. Yoshida et al. suggested the usefulness of ORi for patients in the ICU, when suctioning through an endotracheal tube is performed during ventilation [58]. Furthermore, the fact that the ORi device is noninvasive and easy-to-wear will enable its use in various clinical situations. Recently, COVID-19 has become a worldwide epidemic. ORi might be useful to prevent infections, such as COVID-19, by reducing the frequency of contact of clinical workers with patient blood when performing blood gas analysis or securing an arterial line. The use of ORi will not only enable the early prediction of hypoxemia, but may also be useful for avoiding hyperoxia. In recent years, there have been reports that the mortality rate of patients managed for hyperoxia is high [59]. Thus, in the near future, ORi monitoring may become an effective method to lower the adverse effects caused by high oxygen concentrations. ORi not only determines the optimal inhaled oxygen concentration during the perioperative period, but also acts as an index of proper oxygenation, and has the potential to help avoid complications caused by hypoxemia and hyperoxia. Studies on how the use of ORi can change patient prognoses should be performed in the near future.

## Data Availability

Not applicable.

## References

[1] Miskovic A, Lumb AB (2017). Postoperative pulmonary complications. Br J Anaesth.

[2] Edmark L, Kostova-Aherdan K, Enlund M, Hedenstierna G (2003). Optimal oxygen concentration during induction of general anesthesia. Anesthesiology.

[3] Edmark L, Auner U, Enlund M, Ostberg E, Hedenstierna G (2011). Oxygen concentration and characteristics of progressive atelectasis formation during anaesthesia. Acta Anaesthesiol Scand.

[4] Kiss T, Bluth T, Gama de Abreu M (2016). Does intraoperative lung-protective ventilation reduce postoperative pulmonary complications?. Anaesthesist..

[5] Serpa Neto A, Schultz MJ, Gama de Abreu M (2015). Intraoperative ventilation strategies to prevent postoperative pulmonary complications: systematic review, meta-analysis, and trial sequential analysis. Best Pract Res Clin Anaesthesiol..

[6] Nimmagadda U, Salem MR, Crystal GJ (2017). Preoxygenation: physiologic basis, benefits, and potential risks. Anesth Analg.

[7] De Jong A, Futier E, Millot A, Coisel Y, Jung B, Chanques G, Baillard C, Jaber S (2014). How to preoxygenate in operative room: healthy subjects and situations "At Risk". Ann Fr Anesth Reanim.

[8] Hellström A, Smith LE, Dammann O (2013). Retinopathy of prematurity. Lancet.

[9] Wenk M, Van Aken H, Zarbock A (2017). The New World Health Organization recommendations on perioperative administration of oxygen to prevent surgical site infections: a dangerous reductionist approach?. Anesth Analg.

[10] Damiani E, Adrario E, Girardis M, Romano R, Pelaia P, Singer M, Donati A (2014). Arterial hyperoxia and mortality in critically ill patients: a systematic review and meta-analysis. Crit Care.

[11] Stub D, Smith K, Bernard S, Nehme Z, Stephenson M, Bray JE, Cameron P, Barger B, Ellims AH, Taylor AJ, Meredith IT, Kaye DM, AVOID Investigators (2015). Air versus oxygen in ST-segment-elevation myocardial infarction. Circulation.

[12] Austin MA, Wills KE, Blizzard L, Walters EH, Wood-Baker R (2010). Effect of high flow oxygen on mortality in chronic obstructive pulmonary disease patients in prehospital setting: randomised controlled trial. BMJ.

[13] Kopsaftis Z, Carson-Chahhoud KV, Austin MA, Wood-Baker R (2020). Oxygen therapy in the pre-hospital setting for acute exacerbations of chronic obstructive pulmonary disease. Cochrane Database Syst Rev..

[14] Kilgannon JH, Jones AE, Parrillo JE, Dellinger RP, Milcarek B, Hunter K, Shapiro NI, Trzeciak S, Emergency Medicine Shock Research Network (EMShockNet) Investigators (2011). Relationship between supranormal oxygen tension and outcome after resuscitation from cardiac arrest. Circulation.

[15] Ferguson LP, Durward A, Tibby SM (2012). Relationship between arterial partial oxygen pressure after resuscitation from cardiac arrest and mortality in children. Circulation.

[16] Severinghaus JW (2007). Takuo Aoyagi: discovery of pulse oximetry. Anesth Analg.

[17] Horne C, Derrico D (1999). Mastering ABGs. The art of arterial blood gas measurement. Am J Nurs..

[18] Scheeren TWL, Belda FJ, Perel A (2018). The oxygen reserve index (ORI): a new tool to monitor oxygen therapy. J Clin Monit Comput.

[19] Scheeren TWL, Belda FJ, Perel A (2018). Correction to: the oxygen reserve index (ORI): a new tool to monitor oxygen therapy. J Clin Monit Comput.

[20] Chen ST, Min S (2020). Oxygen reserve index, a new method of monitoring oxygenation status: what do we need to know?. Chin Med J (Engl).

[21] Szmuk P, Steiner JW, Olomu PN, Ploski RP, Sessler DI, Ezri T (2016). Oxygen reserve index: a novel noninvasive measure of oxygen reserve—a pilot study. Anesthesiology.

[22] Hirata N, Chaki T, Yamakage M (2016). Oxygen reserve index: a novel and noninvasive method for monitoring oxygenation (in Japanese with English abstract). J Clin Anesth (Japan)..

[23] Applegate RL, Dorotta IL, Wells B, Juma D, Applegate PM (2016). The relationship between oxygen reserve index and arterial partial pressure of oxygen during surgery. Anesth Analg.

[24] Vos JJ, Willems CH, van Amsterdam K, van den Berg JP, Spanjersberg R, Struys MMRF, Scheeren TWL (2019). Oxygen reserve index: validation of a new variable. Anesth Analg.

[25] Yoshida K, Isosu T, Noji Y, Ebana H, Honda J, Sanbe N, Obara S, Murakawa M (2020). Adjustment of oxygen reserve index (ORi™) to avoid excessive hyperoxia during general anesthesia. J Clin Monit Comput.

[26] Hinkelbein J, Kranke P (2018). Rapid sequence induction. Anästhesiol Intensivmed Notfallmed Schmerzther.

[27] Siddik S, Jalbout M, Baraka AS (2004). Rapid sequence induction for cesarean section. Middle East J Anaesthesiol.

[28] Eichelsbacher C, Ilper H, Noppens R, Hinkelbein J, Loop T (2018). Rapid sequence induction and intubation in patients with risk of aspiration: recommendations for action for practical management of anesthesia. Anaesthesist.

[29] Yoshida K, Isosu T, Noji Y, Hasegawa M, Iseki Y, Oishi R, Imaizumi T, Sanbe N, Obara S, Murakawa M (2018). Usefulness of oxygen reserve index (ORi™), a new parameter of oxygenation reserve potential, for rapid sequence induction of general anesthesia. J Clin Monit Comput.

[30] Miller L, Luković E, Wagener G (2020). Guiding airway management and personal protective equipment for COVID-19 intubation teams. Br J Anaesth.

[31] Abola RE, Schwartz J, Forrester JD, Gan TJ (2021). A practical guide for anesthesia providers on the management of coronavirus disease 2019 patients in the acute care hospital. Anesth Analg.

[32] Begley JL, Lavery KE, Nickson CP, Brewster DJ (2020). The aerosol box for intubation in coronavirus disease 2019 patients: an in-situ simulation crossover study. Anaesthesia.

[33] Rose P, Veall J, Chima N, Vowels E, Chitnis S, Flexman A, Tang R (2020). A comparison of droplet and contact contamination using 3 simulated barrier techniques for COVID-19 intubation: a quality assurance study. CMAJ Open.

[34] Noor Azhar M, Bustam A, Poh K, Ahmad Zahedi AZ, Mohd Nazri MZA, Azizah Ariffin MA, Md Yusuf MH, Zambri A, Chong JYO, Kamarudin A, Ang BT, Iskandar A, Chew KS (2020). COVID-19 aerosol box as protection from droplet and aerosol contaminations in healthcare workers performing airway intubation: a randomised cross-over simulation study. Emerg Med J..

[35] Yoshida K, Isosu T, Imaizumi T, Obara S, Murakawa M (2019). Oxygen reserve index (ORi™) as an alarm for oxygenation deterioration in pediatric tracheostomaplasty: a case report. Paediatr Anaesth.

[36] Lange M, Jonat S, Nikischin W (2002). Detection and correction of endotracheal-tube position in premature neonates. Pediatr Pulmonol.

[37] Yoo SY, Kim JH, Han SH, Oh AY (2007). A comparative study of endotracheal tube positioning methods in children: safety from neck movement. Anesth Analg.

[38] Ishikawa S, Lohser J (2011). One-lung ventilation and arterial oxygenation. Curr Opin Anaesthesiol.

[39] Karzai W, Schwarzkopf K (2009). Hypoxemia during one-lung ventilation: prediction, prevention, and treatment. Anesthesiology.

[40] Koishi W, Kumagai M, Ogawa S, Hongo S, Suzuki K (2018). Monitoring the oxygen reserve index can contribute to the early detection of deterioration in blood oxygenation during one-lung ventilation. Minerva Anestesiol.

[41] Alday E, Nieves JM, Planas A (2020). Oxygen reserve index predicts hypoxemia during one-lung ventilation: an observational diagnostic study. J Cardiothorac Vasc Anesth.

[42] Tsymbal E, Ayala S, Singh A, Applegate RL, Fleming NW (2020). Study of early warning for desaturation provided by oxygen reserve index in obese patients. J Clin Monit Comput..

[43] Ishida Y, Nakazawa K, Okada T, Tsuzuki Y, Kobayashi T, Yamada R, Uchino H (2021). Anesthetic management of a morbidly obese patient with endometrial cancer during robot-assisted laparoscopic surgery. JA Clin Rep.

[44] Martínez G, Cruz P (2008). Atelectasis in general anesthesia and alveolar recruitment strategies. Rev Esp Anestesiol Reanim.

[45] Talab HF, Zabani IA, Abdelrahman HS, Bukhari WL, Mamoun I, Ashour MA, Sadeq BB, El Sayed SI (2009). Intraoperative ventilatory strategies for prevention of pulmonary atelectasis in obese patients undergoing laparoscopic bariatric surgery. Anesth Analg.

[46] Niwa Y, Shiba J, Fujita H, Oka R, Takeuchi M (2019). Oxygen reserve index (ORi™) contributes to prediction of hypoxemia and patient safety during tracheal stent insertion using rigid bronchoscopy: a case report. J Clin Monit Comput.

[47] Fleming NW, Singh A, Lee L, Applegate RL (2021). Oxygen reserve index: utility as an early warning for desaturation in high-risk surgical patients. Anesth Analg.

[48] Lasocki S, Brochant A, Leger M, Gaillard T, Lemarié P, Gergaud S, Dupré P (2019). ORI monitoring allows a reduction of time with hyperoxia in critically ill patients: the randomized control ORI^2^ study. Intensive Care Med.

[49] Kumagai M, Kurihara H, Ishida K, Komatsu H, Suzuki K (2020). The oxygen reserve index as a determinant of the necessary amount of postoperative supplemental oxygen. Minerva Anestesiol..

[50] Habre W, Peták F (2014). Perioperative use of oxygen: variabilities across age. Br J Anaesth..

[51] Kaplan E, Bar-Yishay E, Prais D, Klinger G, Mei-Zahav M, Mussaffi H, Steuer G, Hananya S, Matyashuk Y, Gabarra N, Sirota L, Blau H (2012). Encouraging pulmonary outcome for surviving, neurologically intact, extremely premature infants in the post surfactant era. Chest.

[52] Jobe AH, Bancalari E (2001). Bronchopulmonary dysplasia. Am J Respir Crit Care Med.

[53] Rowland R, Newman CG (1969). Pulmonary complications of oxygen therapy. J Clin Pathol.

[54] Fan Q, Chen M, Fang X, Bond Lau W, Xue L, Zhao L, Zhang H, Liang YH, Bai X, Niu HY, Ye J, Chen Q, Yang X, Liu M (2013). Aging might augment reactive oxygen species (ROS) formation and affect reactive nitrogen species (RNS) level after myocardial ischemia/reperfusion in both humans and rats. Age (Dordr).

[55] Fan Q, Chen L, Cheng S, Fang L, Lau WB, Wang LF, Liu JH (2014). Aging aggravates nitrate mediated ROS/RNS changes. Oxid Med Cell Longev.

[56] Isosu T, Yoshida K, Oishi R, Imaizumi T, Iseki Y, Sanbe N, Ikegami Y, Obara S, Kurosawa S, Murakawa M (2018). Effects of indigo carmine intravenous injection on oxygen reserve index (ORi™) measurement. J Clin Monit Comput.

[57] Kondo H, Nakamura R, Kobatake A, Nao Y, Hashimoto K, Nakatani K (2020). Effects of intravenous injection of indocyanine green on the oxygen reserve index (ORi™). J Anesth.

[58] Yoshida K, Isosu T, Murakawa M (2018). Making use of the oxygen reserve index (ORi™): a new parameter of oxygenation reserve potential. Pulm Crit Care Med.

[59] Pala Cifci S, Urcan Tapan Y, Turemis Erkul B, Savran Y, Comert B (2020). The impact of hyperoxia on outcome of patients treated with noninvasive respiratory support. Can Respir J.

